# The impact of early rehabilitation program on exercise tolerance in post-myocardial infarction patients: a 5-week intervention study

**DOI:** 10.1186/s12872-025-04988-y

**Published:** 2025-07-25

**Authors:** Agnieszka Grochulska, Aleksandra Bryndal, Sebastian Glowinski

**Affiliations:** 1 Department of Physiotherapy, Institute of Health Sciences, Pomeranian University in Slupsk, Westerplatte 64, Slupsk, 76-200 Poland; 2 Institute of Physical Culture and Health, State Academic of Applied Sciences in Koszalin, Lesna 1, Koszalin, 75-582 Poland

**Keywords:** Myocardial infarction, Cardiac rehabilitation, Exercise tolerance, Cardiorespiratory fitness, Cardiovascular remodeling, Hemodynamics, Physiotherapy

## Abstract

**Background:**

Cardiovascular diseases remain the leading cause of death worldwide, with cardiac rehabilitation playing a key role in recovery after myocardial infarction. This study aimed to assess the impact of an early 5-week cardiac rehabilitation program (24 training sessions; 5 sessions/week), implemented approximately 17 days after myocardial infarction, on improving exercise tolerance.

**Methods:**

The study included 188 patients (133 men, 55 women, mean age 61.36 years) with first ST-segment elevation myocardial infarction after successful revascularization. The rehabilitation program consisted of general conditioning exercises, endurance training, and resistance training. Assessment was performed before and after rehabilitation using electrocardiographic exercise testing, 6-minute walk test, and hemodynamic measurements.

**Results:**

After the 5-week program, significant improvements were observed in maximum heart rate (113.89 ± 14.90 to 121.27 ± 15.91 beats/min, *p* = 0.0001), physical capacity expressed in metabolic equivalent of task (METs) (6.28 ± 1.98 to 8.50 ± 2.64, *p* = 0.0001), double product reserve (DPr) (18,063.48 ± 6,531.58 to 19,115.82 ± 4,021.28, *p* = 0.0001), and distance in 6-minute walk test (483.46 ± 105.15 to 535.53 ± 98.47 m, *p* = 0.0001). A significant decrease in peak diastolic blood pressure (80.03 ± 7.32 to 78.56 ± 7.44 mmHg, *p* = 0.04) and perceived exertion on the Borg scale (14.06 ± 1.90 to 13.05 ± 0.92, *p* = 0.0001) was also noted.

**Conclusions:**

Early, intensive cardiac rehabilitation leads to significant improvement in exercise tolerance after just 5 weeks, as demonstrated by changes in key cardiovascular parameters. Implementing intensive, short-term rehabilitation programs during the critical period of cardiac remodeling may provide an effective alternative to longer programs, particularly in healthcare systems with limited resources.

**Supplementary Information:**

The online version contains supplementary material available at 10.1186/s12872-025-04988-y.

## Introduction

Cardiovascular diseases remain the leading cause of death worldwide, accounting for approximately 20.5 million deaths annually (data from 2021). About 80% of all CVD-related deaths occur in low- and middle-income countries. Ischemic heart disease and stroke account for 85% of deaths associated with cardiovascular diseases [1]. It is projected that by 2050, there will be an increase in the prevalence of cardiovascular diseases with predictions of 35.6 million deaths from cardiovascular causes in 2050 [2].

The World Health Organization estimates that 75% of cardiovascular mortality can be reduced through the implementation of appropriate lifestyle changes, with cardiac rehabilitation playing a key role in this prevention strategy [3]. Cardiac rehabilitation (CR) in Poland has shown significant clinical benefits for patients with cardiovascular diseases, especially those recovering from myocardial infarction (MI). The Coordinated Cardiac Care Program after Myocardial Infarction (KOS-MI) demonstrated a significant reduction in major adverse cardiac and cerebrovascular events (MACCE), namely a 50% reduction in overall MACCE, a 59% reduction in mortality rates, a 39% reduction in recurrent myocardial infarction, a 40% reduction in repeat revascularization, and a 39% reduction in hospitalizations for heart failure [4].

Numerous studies confirm that only a small percentage of patients are referred to centers providing comprehensive cardiac rehabilitation (CR). According to Smith et al. (2023), despite the proven benefits of cardiac rehabilitation, participation in CR programs in Europe ranges from as low as 10–60%, with significant differences between countries, and an average of approximately 30% [5]. Research by Abreu et al. demonstrated that in European countries, on average, only 41% of eligible patients after myocardial infarction are referred to cardiac rehabilitation, of which only 34% ultimately participate [6]. The situation in Poland is even more concerning - according to the European Society of Cardiology (ESC) report from 2022, in Poland, only about 22% of patients after acute coronary events participate in a full cardiac rehabilitation program [7]. A study by Salzwedel et al. (2024) indicates that despite the implementation of Coordinated Care Programs in Poland, still less than 30% of patients after myocardial infarction complete a full cycle of cardiac rehabilitation [8]. Studies by Grace confirm that despite improvements in recent years, there remains a large discrepancy in Poland between the number of patients eligible for cardiac rehabilitation and the number of people who actually participate, estimated at 25–35% [9]. The main reasons for this situation are primarily differences in healthcare organization and the costs of medical procedures. The excessively low valuation of healthcare services results in poor profitability for healthcare providers [10]. Meanwhile, risk factors for cardiovascular events such as lack of physical activity or insufficient activity can be modified. However, much depends on the awareness of both physicians and patients about the benefits of exercise and willingness to undertake effort despite certain physical limitations [11].

Cardiac rehabilitation programs according to various international guidelines consist of several phases of different durations. Currently, in European countries, the duration of comprehensive cardiac rehabilitation programs is 8–12 weeks (2–3 sessions/week, not consecutive) [6]. When implemented in inpatient settings, it lasts 3–4 weeks (6 sessions/week) [6, 16]. In Poland, the National Health Fund reimburses a 24-day rehabilitation program for outpatient facilities (24 training sessions within 90 calendar days), which can be implemented as 3–5 sessions per week [17]. The frequency of training sessions sufficient to induce significant physiological effects is 3–5 times per week, while fewer than 3 sessions weekly will not significantly contribute to achieving the intended goals [12, 13].

Cardiac rehabilitation programs can vary in length, and studies suggest that both 6-week and 12-week programs offer benefits, but with certain key differences. Cardiac rehabilitation is typically conducted over a period of 12 weeks. Shorter programs (4–6 weeks) with high-frequency sessions may provide similar benefits [14]. Although specific frequencies of training sessions per week are recommended in different countries, research emphasizes that cardiac rehabilitation should be tailored to the individual needs of the patient. The main goal is to improve cardiovascular exercise tolerance, functional fitness, and quality of life, rather than strictly adhering to a rigid number of sessions. The scientific community continues to investigate optimal rehabilitation strategies, recognizing that exercise frequency may need to be adjusted to specific cardiovascular conditions and individual patient characteristics and capabilities [15]. Currently, there is no universal agreement regarding when hemodynamic changes improving cardiorespiratory fitness are observed earliest under the influence of implemented cardiac rehabilitation programs, and there are divided opinions regarding the frequency of training sessions, namely whether 2–3 times per week or 5 times per week is optimal [14, 15, 17].

The aim of this study is to assess the impact of a 5-week (24 training sessions; 5 sessions/week) early cardiac rehabilitation program implemented within two weeks after completed treatment of the acute phase of the disease on improving exercise tolerance by the cardiovascular system in patients after myocardial infarction in an outpatient setting. Hypothesis: A 5-week early rehabilitation program containing 24 training sessions increases exercise tolerance by the cardiovascular system.

## Materials and methods

### Participants

The study was conducted from April 2019 to June 2023 among 188 patients after myocardial infarction. Patients participated in the Coordinated Specialist Care Program - Infarct (CSC-Infarct) at the Cardiac Rehabilitation Center of the Regional Specialist Hospital in Słupsk, to which they were admitted on average on day 17 (range 9.0–26.0) post-myocardial infarction, corresponding to up to 14 days after hospital discharge. Purposive sampling was used for sample selection. A total of 188 individuals were examined, including 55 women (29.26%) and 133 men (70.74%).

### Selection criteria

The following inclusion and exclusion criteria were applied in the study. Inclusion criteria were: referral for comprehensive cardiac rehabilitation in outpatient form implemented up to 14 days after hospital discharge following completed of a first-time ST-segment elevation myocardial infarction, after complete, simultaneous, successful, uncomplicated revascularization of the infarct-related artery in the acute phase of the coronary incident with achieved flow, with a stable clinical picture, no recurrence of angina symptoms in the first stage of rehabilitation, negative result of initial symptom-limited exercise test, absence of left ventricular heart failure symptoms, without significant complex cardiac rhythm and conduction disorders, controlled hypertension, well-controlled diabetes, age above 18 years, exercise tolerance not less than 3 METs during electrocardiographic exercise testing, and informed consent of the patient to participate in the study.

Exclusion criteria were: ST-segment elevation myocardial infarction within 7 days (according to American Heart Association recommendations), symptomatic severe aortic stenosis, left coronary artery stenosis, unstable angina, acute pulmonary embolism or pulmonary infarction, uncompensated heart failure, deep vein thrombosis, mobile or fresh thrombus in the heart chambers, myocarditis, endocarditis or pericarditis, or aortic dissection, symptomatic second and third-degree atrioventricular block without pacemaker protection (acquired), poorly controlled hypertension, recent stroke or cerebral ischemia, other acute or uncompensated non-cardiac disease that could interfere with the performance and result of electrocardiographic exercise testing, as well as age under 18 years, exercise tolerance less than 3 METs during electrocardiographic exercise testing, and lack of informed consent from the patient to participate in the study.

### Instruments

Patients began rehabilitation on average 17 days (± 3; range 9–26) after myocardial infarction and up to 14 days after hospital discharge.

During qualification for the comprehensive cardiac rehabilitation program, a symptom-limited exercise test on a treadmill with electrocardiographic monitoring was performed, along with a standard 12-lead electrocardiographic recording, 6-minute walk test (6MWT), echocardiographic examination, waist-to-hip ratio (WHR), waist circumference measurement, assessment of double product reserve (DPr), and BMI. After completion of the 5-week (24 training sessions; 5 sessions/week) cardiac rehabilitation program, the treadmill exercise test with ECG and 6MWT were repeated [18].

The electrocardiographic exercise test was performed on an ASTEL treadmill with continuous monitoring of 12 electrocardiogram leads, using a symptom-limited test according to the Bruce protocol, gradually increasing the load every 3 min by accelerating the belt speed and increasing the treadmill incline angle. Blood pressure was measured using a clock sphygmomanometer by the Korotkoff method before starting the test, in the third minute of each load stage, at the moment of exercise completion, and at 1, 3, and 6 min after exercise completion.

The following parameters were analyzed: resting heart rate (HR rest), peak exercise heart rate (HR max), and heart rate at 1 min after completed exercise (1-minute heart rate recovery − 1-minute HRR), expressed in beats/minute, and metabolic equivalent of task (MET), which is the estimated energy cost of a given activity divided by resting energy expenditure (1 MET = 3.5 ml O₂/kg/min of oxygen consumption VO₂). Resting systolic (SBP rest) and diastolic (DBP rest) blood pressure values and peak systolic (SBP peak) and diastolic (DBP peak) blood pressure values at peak exercise, expressed in mmHg, were also analyzed. The double product reserve (DPr) was calculated as the product of heart rate (HR max) and systolic blood pressure (SBP peak) at peak exercise, which is an indirect indicator of myocardial oxygen demand, and its peak value can be used as one of the parameters in assessing exercise capacity of cardiac workload during functional tests.

The criteria for terminating the electrocardiographic exercise test were: patient-reported chest pain, dizziness, ataxia, dyspnea, fatigue without signs of myocardial ischemia, reaching the target heart rate, ST-segment depression > 1 mm in leads without pathological Q wave or ST-segment depression > 2 mm horizontally or downsloping, detection of new segmental wall motion abnormalities, occurrence of complex supraventricular arrhythmias, blood pressure decrease > 10 mmHg compared to baseline with accompanying signs of ischemia, blood pressure increase > 240/110 mmHg, or lower limb pain [18, 19].

The 6-minute walk test (6MWT) was performed in a 30-meter corridor. The walking distance was marked with strips, and distance markers were placed every 3 m. A stopwatch, pulse oximeter, and medical sphygmomanometer were used in the study. Before starting the test, the subject rested in a sitting position for 10 min. Patients were also advised against intense physical exercise and drinking stimulating beverages 2 h before starting the test. Patients were instructed to walk at their own pace during the test and slow down if necessary. The aim of the test was to assess walking distance, heart rate, blood pressure, blood oxygen saturation, and fatigue using the 20-point modified Borg scale [21]. In accordance with American Thoracic Society (ATS) guidelines [22], the criterion for ending the test was reaching 6 min. Criteria for interrupting the test were: patient-reported chest pain, significant dyspnea, fatigue, concerns of “intermittent claudication” (calf pain), balance disorders, pallor, cold sweats, or a decrease in blood oxygen saturation below 85% SpO2. All patients completed the 6MWT. No clinical complications were recorded during the tests or within 5 h after test completion [20].

The tests were conducted in the morning hours, in an air-conditioned room, at an ambient temperature of 18–22 °C and humidity of 40–60%. On the day of testing, patients took their regular medications and did not consume stimulating beverages (e.g., coffee, cola), smoke cigarettes, or perform physical exercises.

Another examination performed was an echocardiographic study using an Acuson 128 device with a transthoracic 3.5 MHz probe. Left ventricular ejection fraction (LVEF/EF) was assessed using the Simpson method in accordance with the current recommendations of the American Society of Echocardiography, with the average of measurements in three cardiac cycles considered in the assessment [23].

Somatic measurements were performed, including height and weight, waist and hip circumferences, based on which body proportion indices were calculated. The body mass index (BMI) was calculated for each subject. BMI was calculated according to the following formula: weight in kilograms divided by height expressed in meters squared. Based on this, patients were classified as underweight (under 18.5), normal weight (18.5–24.9), pre-obesity (25.0–29.0), or obesity (above 30.0) [24]. The waist-to-hip ratio (WHR), which reflects the distribution of adipose tissue with characteristic placement of excessively developed adipose tissue in the abdomen and hips, allows for determining the type of body shape predisposing to cardiovascular diseases. Waist circumference < 80 cm (women) and < 94 cm (men) are considered normal parameters [25]. Both indices were analyzed by gender, and their relationship with the age of the subjects was examined.

Medical history regarding comorbidities was also collected from patients, and data analysis was performed for the presence of major coronary heart disease risk factors such as gender, diabetes, hypertriglyceridemia, hypertension, smoking, and obesity.

The studies were conducted in accordance with ethical requirements. Each patient provided informed and written consent to participate and was informed about the course and purpose of the study. Each patient also consented to the use and processing of their medical data.

### Procedure

Patients began the rehabilitation process on average on day 17 (± 3; range 9–26) after undergoing complete, simultaneous, successful, uncomplicated revascularization, which corresponded to up to 14 days after hospital discharge. Outpatient cardiac rehabilitation lasted 5 weeks comprising 24 training sessions, which took place 5 days a week, followed by a 2-day break.

Patients qualified for the study were under pharmacological treatment according to the standards of the European Society of Cardiology (ESC) and did not require modification of this treatment during the cardiac rehabilitation program.

The physical rehabilitation program was developed according to the FITT principle (frequency, intensity, time-duration, type of exercise) [17, 26]. Determination of physical exercise intensity was conducted according to the guidelines of the Cardiac Rehabilitation and Exercise Physiology Section of the Polish Cardiac Society, which published recommendations for qualification to one of the rehabilitation models: A, B, C, or D based on the results of the electrocardiographic treadmill exercise test and cardiovascular event risk assessment (Table 1).

**Table 1:** Rehabilitation models based on initial cardiovascular exercise tolerance from the preliminary electrocardiographic exercise test

Rehabilitation Model	Cardiac Event Risk Stratification Model and Exercise Tolerance	Type of exercise, frequency, time -duration	Intensity
Model **A**	Patients with low risk and good exercise tolerance (> 7 METs)	**Endurance Training** • Continuous type with constant intensity − 5 times per week, in 30-minute sessions **Resistance Training** • 2–3 times per week, in 15-minute sessions **General Conditioning Exercises** • 5 times per week in 45-minute sessions	**Intensity** 60–80% of heart rate reserve or 50–70% of maximum workload.
**Model B**	Patients with moderate risk and moderate exercise tolerance (> 5 METs) and with moderate risk and high exercise tolerance (> 7 METs)	**Endurance Training** • Continuous type with constant intensity − 5 times per week, in 30-minute sessions (for patients with good exercise tolerance) • Continuous type with variable intensity − 5 times per week, in 30-minute sessions (for patients with moderate exercise tolerance) **Resistance Training** • 2–3 times per week, in 15-minute sessions **General Conditioning Exercises** • 5 times per week, in 30-minute sessions	**Intensity** 50–60% of heart rate reserve or 50% of maximum workload.
**Model C**	Patients with moderate risk and low exercise tolerance (3–5 METs) and with high risk and good exercise tolerance (> 6 METs)	**Endurance Training** • Continuous type with constant intensity − 2 times a week, in 5–10 min sessions • Continuous type with variable intensity − 5 times a week, in 20-minute sessions **Resistance Training** • 2–3 times a week, in 15-minute sessions **General Fitness Exercises** • 5 times a week, in 30-minute sessions	**Intensity** 40–50% of heart rate reserve or 40–50% of maximum load
**Model D**	Patients with moderate risk and very low physical exercise tolerance (< 3 METs) and high risk, moderate, low, and very low physical exercise tolerance (< 6 METs)	**Individually Tailored Exercises** 2–3 times daily for 3–5 days a week, in 30–45 min sessions	**Intensity** Below 20% of heart rate reserve or below an increase of 10–15% of resting heart rate

Source: Own elaboration based on Recommendations for the Implementation of Comprehensive Cardiac Rehabilitation, Section of Cardiac Rehabilitation and Exercise Physiology of the Polish Cardiac Society (29)

In assessing the risk of cardiovascular events, the clinical diagnosis, left ventricular systolic function, complex ventricular arrhythmias, signs of myocardial ischemia observed during electrocardiographic exercise testing, physical exercise tolerance, and hemodynamic responses to exercise were considered, and classified as low, moderate, or high risk. Patients who meet al.l criteria are classified in the low-risk group. The presence of one criterion of moderate or high risk would classify the patient into the respective group. Low risk: LVEF > 50%, absence of complex ventricular arrhythmias and ischemia on ECG, exercise tolerance > 7 MET/100 W, normal hemodynamic response, clinically - uncomplicated infarction, no critical coronary stenoses. Moderate risk: LVEF 36–49%, ST-segment depression of 1–2 mm, exercise tolerance 5-6.9 MET/75–100 W, clinically - uncomplicated infarction. High risk: LVEF < 35%, presence of complex ventricular arrhythmias, ST-segment depression > 2 mm, exercise tolerance < 5 MET/75 W, abnormal hemodynamic response, presence of critical coronary stenoses, clinically - infarction complicated by shock or heart failure [27]. The absence of a control group in this study was primarily due to limited access to participants, which made it unfeasible to recruit a sufficient number of controls. While this limitation restricts the ability to draw direct comparisons, we believe the findings provide valuable insights. Future research would benefit from randomized controlled trials (RCTs) to further validate these results and strengthen the conclusions.

After conducting initial physical and medical examinations and qualifying for the appropriate cardiac rehabilitation model, patients underwent physiotherapeutic instruction on proper exercise performance, safety rules during the rehabilitation program, and independent monitoring of basic physiological parameters, such as heart rate (HR). During the comprehensive cardiac rehabilitation program, patients participated in four educational meetings held once a week for 30 min, focusing on providing dietary support aimed at reducing atherosclerosis risk factors. The rehabilitation program also included eight psychological interventions held twice a week for 30 min, addressing self-assessment of disease exacerbation symptoms, returning to recreational, professional, and sexual activities, as well as coping with anxiety, depression, and improving cognitive functions. Patients were presented with information about the consequences of an inappropriate lifestyle and non-adherence to recommended pharmacological treatment. Additionally, participants were enrolled in weekly behavioral-relaxation therapy sessions, which included: learning relaxation techniques, modifying personal life philosophy, personal attitude towards life and motivation, elements of emotional support, improving self-esteem, and learning stress management strategies [28–31].

The kinesiotherapeutic cardiac rehabilitation program included all 188 patients and consisted of general fitness exercises, endurance training on a cycle ergometer, and treadmill walking training under the supervision of a physiotherapist, with constant monitoring of the hemodynamic circulation response and patient self-assessment using the Borg scale [12, 27].

General fitness training consisting of a warm-up lasting 5 min, general fitness training lasting 30 min, and a cool-down lasting 5 min. The 40-minute general fitness exercise program was conducted in a gymnasium and included: active exercises with and without equipment (gymnastics sticks, balls), active resistance exercises (bands, dumbbells), coordination, relaxation, breathing, and isotonic exercises. Controlled breathing exercises were implemented, aimed at improving respiratory system efficiency and learning proper breathing. During inhalation, efforts were made to increase diaphragm work and activate the lower rib cage. Particular emphasis was placed on the method of performing exercises, paying attention to the correct breathing rhythm and extending the exhalation phase. All exercises were performed with a gradual change in body position, starting from low positions (lying on the back), then half-high positions (sitting), and ending with high positions (standing) [12, 27].

Resistance training engaging various muscle groups was conducted 3 times a week. Individual exercises were synchronized with breathing (resistance during exhalation), performed slowly, adhering to the full range of motion. Initial load was adjusted so that the patient could perform 12–15 repetitions of a given exercise, engaging approximately 30-50% of maximum muscle strength. Exercise sets were performed 1–3 times, with breaks between sets lasting 30–60 s. Exercises were performed at an intensity perceived by the patient at 11–13 points on the Borg scale [12, 27].

Endurance training of a continuous type with variable intensity, or of a continuous type with constant intensity on a bicycle ergometer, was performed depending on qualification for the appropriate cardiac rehabilitation model A, B, C, or D (Table 1) [12, 27].

Endurance training of a continuous type with variable intensity consisted of 5 work and rest cycles. A single interval included a 4-minute work period of cycling on an ergometer with increasing load, starting from 20 W, increasing by 10–20 W in subsequent cycles until reaching the target load and heart rate (training heart rate), followed by a 1-minute work period of cycling on an ergometer with minimal load of 5 W, necessary to avoid adverse hemodynamic effects of sudden cessation of physical exertion. Heart rate (HR) and blood pressure (BP) measurements were taken at rest, then at the peak of exertion during the training and 1 min after completing physical exercise (1 min HRR). Additionally, after completion, the patient performed self-assessment of fatigue using the Borg scale.

Endurance training of a continuous type with constant intensity was conducted according to the following scheme: 3 min warm-up, 25 min main training, 2 min cool-down. Total training time on the ergometer was 30 min. Cycling on the ergometer with increasing load during the warm-up phase, until reaching the target load and training heart rate in the main training phase, followed by a 2-minute work period of cycling on the ergometer with decreasing load in the cool-down phase. Training was conducted under constant ECG monitoring. Heart rate (HR) and blood pressure (BP) measurements were taken at rest, then every 6 min during training and 1 min after completing physical exercise (1 min HRR). Additionally, after completing the training, the patient performed self-assessment of fatigue using the Borg scale [12, 27]. Endurance training was conducted 5 times a week under the supervision of a physiotherapist, with constant monitoring of the hemodynamic circulation response and patient self-assessment using the Borg scale. All training sessions were conducted in the morning, in an air-conditioned room at a temperature of 18–22 °C and humidity of 40–60%, in groups of 5 people.

### Statistical analysis

Statistical analysis was performed using Statistica 13.3 software. Quantitative variables were characterized using arithmetic mean, standard deviation, median, minimum and maximum values (range), and 95%CI (confidence interval). Qualitative variables were presented using numerical and percentage values. Initially, the Grubbs test was used to identify outliers (erroneous values). Next, the normal distribution of the variable was examined. For this purpose, the following tests were used: Shapiro-Wilk, Lillieforsa, Kolmogorov-Smirnov, and Jarque-Bera tests. Levene’s test was used to check hypotheses about equal variances. The significance of differences between two groups (unrelated variables model) was investigated using significance difference tests: Student’s t-test (in the case of lack of variance homogeneity) or the Mann-Whitney U test (when t-test applicability conditions were not met). The significance of differences between the same variable in different structures in the absence of a normal distribution was examined using the Kruskal-Wallis test, and in the case of a statistically significant result, an additional post-hoc test was used. In the case of statistically significant results obtained from the Kruskal-Wallis test, post-hoc analyses were conducted with appropriate correction for multiple comparisons to control for the type I error rate. For dependent variables, Wilcoxon’s paired test was also used (when appropriate conditions were met). For qualitative variables, Chi-square NW, Pearson’s Chi-square, and Fisher’s exact tests were used. Before proceeding with the analysis of interdependencies between variables, plots were created illustrating the strength and direction of relationships between them. This allowed for determining whether outliers were present. In all calculations, a significance level of *p* = 0.05 was adopted. Additionally, p-value was included on the graphs.

## Results

### Study group characteristics

**Fig. 1:**
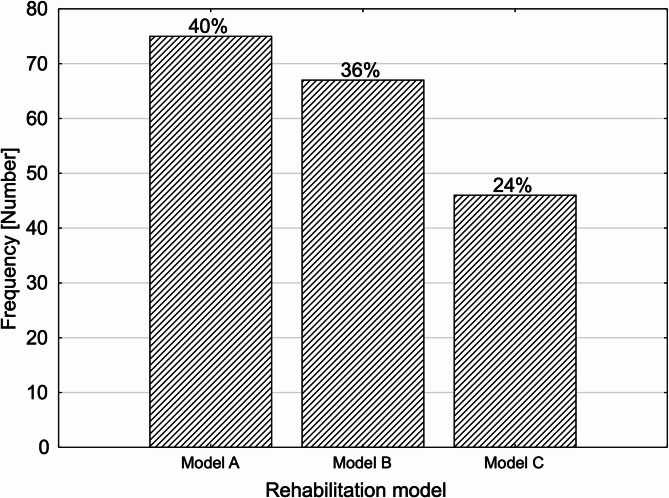
Division of participants into three groups based on the rehabilitation model

Table 2 presents the demographic and clinical characteristics of the study group, consisting of 188 participants. The data include variables such as sex distribution, age, body mass index (BMI), waist-hip ratio (WHR), waist circumference, left ventricular ejection fraction (LVEF/EF), and the time elapsed between myocardial infarction and the start of rehabilitation. Additionally, the study classifies participants according to different rehabilitation models. The study group comprises 29,26% women (55 individuals) and 70.74% men (133 individuals). The mean age of participants is 61.36 years, with a range spanning from 35 to 87 years. The BMI values indicate a tendency toward overweight, with a mean of 28.50 and values ranging between 17.3 and 40.6. The waist-hip ratio (WHR) has a mean of 1.02, with individual values varying from 0.65 to 1.40. Waist circumference measurements show an average of 103.71 cm, reflecting considerable variation among participants. Cardiac function, assessed through left ventricular ejection fraction (LVEF/EF), varies significantly, with a mean of 30.31% and a broad range from 0.30 to 65.00%. Furthermore, the study records the number of days between myocardial infarction and the start of rehabilitation, with a mean of 17.33 days, ranging from 9 to 26 days. Figure 1 presents the rehabilitation model classification and divides participants into three groups: Model A (39.89%), Model B (35.64%), and Model C (24.47%). This categorization helps in analyzing different rehabilitation approaches and their potential impact on recovery.

**Table 2:** Characteristics of the study group

		All group (*N* = 188)
Sex	Number (% from all group)	Women − (55 (29.26%)
		Men – 133 (70.74%)
Age [years]	Mean (SD) Range Me [95% CI]	61.36 (9.67) 35.0–87.0 63.0 [59.97; 62.75]
BMI		28.50 (4.77) 17.3–40.6 28.0 [27.81; 29.18]
WHR - hip-waist ratio		1.02 (0.15) 0.65–1.40 1.0 [1.00; 1,04]
Waist circumference		103.71 (13.82) 64.0-138.0 105.0 [101.72; 105.70]
LVEF/EF (%)		30.31 (25.45) 0.30–65.00 43.0 [26.65; 33.97]
Number of days after myocardial infarction and the start of rehabilitation		17.33 (3.14) 9.0–26.0 17.0 [16.88; 17.78]
Rehabilitation model	Number (% z danej grupy)	A – 75 (39.89%) B – 67 (35.64%) C – 46 (24.47%)

**Fig. 2:**
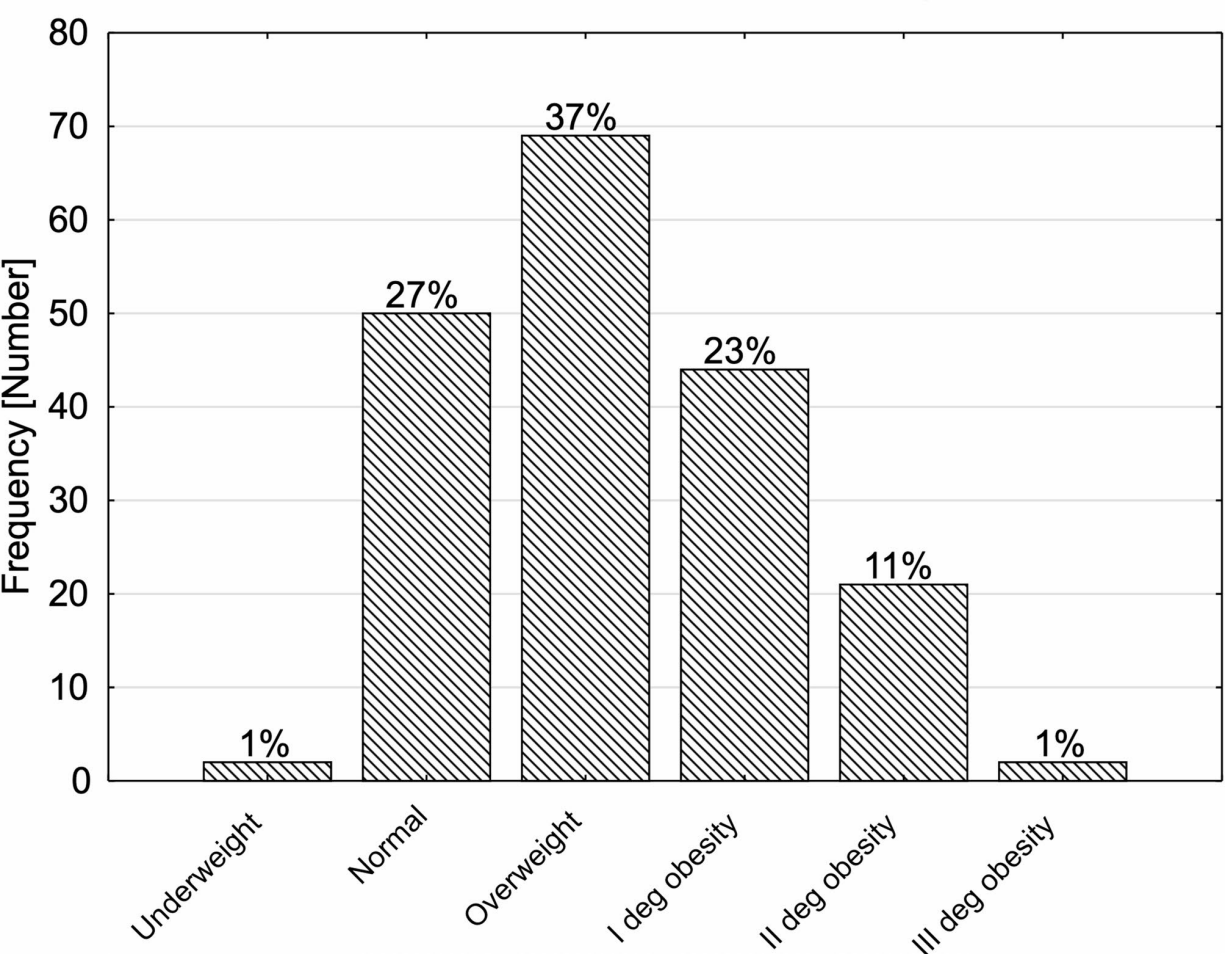
BMI classification of the participants

Table 3 presents data on the occurrence of key health conditions within the study group, including overweight, obesity, hypertriglyceridemia, nicotinism, hypertension, and diabetes. These factors are crucial in assessing the overall cardiovascular risk profile of the participants. Regarding body weight classification, the study identifies that 2 participants (1.06%) were underweight, while 50 individuals (26.60%) had a normal weight. A significant proportion of the group was classified as overweight (69 participants, 36.70%), while varying degrees of obesity were also observed: 44 individuals (23.40%) had first-degree obesity, 21 participants (11.11%) had second-degree obesity, and 2 individuals (1.06%) were classified as having third-degree obesity (Fig. 2). Hypertriglyceridemia was present in the majority of the group, with 144 participants (76.60%) affected, while 44 individuals (23.40%) did not exhibit elevated triglyceride levels. The study also assessed nicotinism (smoking habits) among participants. A significant portion of the study group, 93 individuals (49.46%), reported being smokers, while 95 participants (50.53%) were non-smokers. Hypertension (NT - hypertension) was highly prevalent, affecting 140 participants (74.47%), whereas 48 individuals (25.53%) did not have hypertension. Finally, diabetes mellitus (DM) was present in 73 participants (38.83%), while 115 individuals (61.17%) did not have a diabetes diagnosis.

**Table 3:** Characteristics of the study group in terms of the occurrence of overweight, obesity, hypertriglyceridemia, nicotinism, NT- hypertension, DM - diabetes

Parameter	Overweight, Obesity	Hypertriglyceridemia	Nicotinism	NT- Hypertension	DM - diabetes
Number (% from all group)	Underweight − 2 (1.06) Normal − 50 (26.60) Overweight − 69 (36.70) I deg obesity − 44 (23.40) III deg obesity − 21 (11.17) III deg obesity − 2 (1.06)	Yes 144 (76.60) No 44 (23.40)	Yes 93 (49.46) No 95 (50.53)	Yes 140 (74.47) No 48 (25.53)	Yes 73 (38.83) No 115 (61.17)

### Comparison of parameters before and after 5-week cardiac rehabilitation

**Fig. 3:**
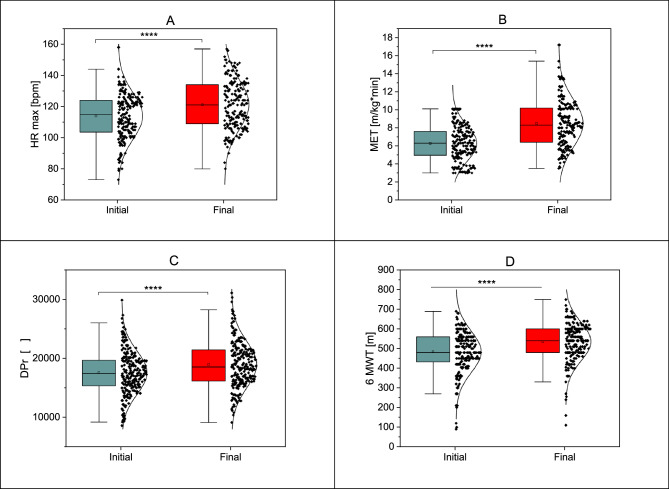
Statistically significant differences in parameters: Initial - Final max HR (3 **A**); Initial - Final MET (3**B**); Initial - Final DPr (3 **C**); Initial - Final 6MWT (3**D**); ****p < 0.0001

**Fig. 4:**
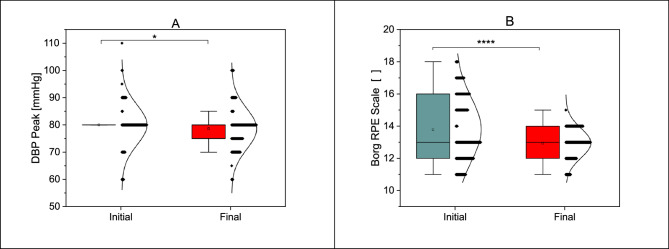
Statistically significant differences in parameters: Initial - Final DBP peak (4 **A**); Initial - Final Borg (4**B**); *p < 0.05; ****p < 0.0001

Table 4 presents the comparison of key physiological parameters before and after the five-week cardiac rehabilitation program (24 training sessions, 5 sessions per week) revealed several significant improvements in patients recovering from myocardial infarction. A notable increase was observed in maximal heart rate (HR max), rising from 113.89 (± 14.90) bpm to 121.27 (± 15.91) bpm (*p* = 0.0001) (Fig. 3A). Additionally, a significant decrease was observed in peak diastolic blood pressure (DBP peak), which reduced from 80.03 (± 7.32) mmHg to 78.56 (± 7.44) mmHg (*p* = 0.0400). One of the most striking changes was the improvement in metabolic equivalent of task (MET), which increased from 6.28 (± 1.98) to 8.50 (± 2.64) (*p* = 0.0001) (Fig. 3B). Similarly, double product (DPr), a measure of myocardial workload, significantly increased from 18,063.48 (± 6,531.58) to 19,115.82 (± 4,021.28) (*p* = 0.0001). Furthermore, the six-minute walk test (6MWT) distance significantly improved from 483.46 (± 105.15) meters to 535.35 (± 98.47) meters (*p* = 0.0001) (Fig. 3D). On the other hand, some parameters remained unchanged, such as resting heart rate (HR rest), one-minute heart rate recovery (HRR), and resting systolic and diastolic blood pressure (SBP rest and DBP rest), all of which showed no statistically significant differences (Fig. 4A). Interestingly, the Borg scale, which assesses perceived exertion, decreased significantly from 14.06 (± 1.90) to 13.05 (± 0.92) (*p* = 0.0001) (Fig. 4B).

**Table 4:** Comparison of parameters before and after 5-week (24 training sessions; 5 sessions/week) cardiac rehabilitation

		All group (*N* = 188)		*p*-value
HR rest	Mean (SD) Range Me [95% CI]	70.93 (11.36)	71.93 (10.90)	0.4845^1^
		47.0-117.0	50.0-109.0	
		70.0	70.5	
		[69.29; 72.56]	[70.36; 73.49]	
HR max		113.89 (14.90)	121.27 (15.91)	**0.0000** ^1^
		73.0-158.0	80.0-157.0	
		115.0	121.0	
		[111.75; 116.03]	[118.98; 123.56]	
1 min HRR		88.63 (13.72)	86.88 (19.33)	0.5711^1^
		55.0-143.0	46.0-135.0	
		88.0	89.0	
		[86.66; 90.61]	[84.10; 89.66]	
SBP rest		122.47 (15.80)	122.45 (16.18)	0.9991^1^
		90.0-170.0	85.0-160.0	
		120.0	120.0	
		[120.20; 124.75]	[120.12; 124.78]	
DBP rest		76.28 (7.84)	76.36 (7.73)	0.8776^1^
		60.0-100.0	60.0-100.0	
		80.0	80.0	
		[75.15; 77.40]	[75.24; 77.47]	
SBP peak		155.56 (20.97)	157.37 (22.49)	0.1951^1^
		95.0-210.0	16.0-230.0	
		160.0	160.0	
		[152.54; 158.58]	[154.14; 160.61]	
DBP peak		80.03 (7.32)	78.56 (7.44)	0.0400^1^
		60.0-110.0	60.0-100.0	
		80.0	80.0	
		[78.97; 81.08]	[77.49; 79.63]	
MET		6.28 (1.98)	8.50 (2.64)	0.0000^1^
		3.0-10.1	3.5–17.2	
		6.3	8.3	
		[6.00; 6.57]	[8.12; 8.88]	
DPr		18063.48 (6531.58)	19115.82 (4021.28)	0.0000^1^
		8600.0-92150.0	10400.0-31120.0	
		17600.0	18735.0	
		[17123.75; 19003.22]	[18537.26; 19694.39]	
6MWT		483.46 (105.15)	535.53 (98.47)	0.0000^1^
		90.0-690.0	110.0-750.0	
		480.0	540.0	
		[468.33; 498.59]	[521.53; 549.70]	
Borg		14.06 (1.90)	13.05 (0.92)	**0.0000** ^1^
		11–18	11–16	
		14	13	
		[13.82; 14.31]	[12.93; 13.17]	

^1^test par Wilcoxona

## Discussion

In this study, the impact of a 5-week early cardiac rehabilitation program was evaluated, in which improvements in physical exercise tolerance by the cardiovascular system can already be observed in patients after myocardial infarction, implemented in an outpatient setting.

After an acute coronary event, the heart muscle undergoes a remodeling process that leads to the formation of a post-infarction scar, typically lasting several weeks. Within 1–3 days of the acute incident, inflammatory cells accumulate in the ischemic area. Subsequently, the inflammatory process subsides, enabling tissue reconstruction and scar formation over the following weeks [32]. Heart remodeling after myocardial infarction (MI) is a complex and dynamic process that depends on multiple factors, including the extent of myocardial infarction, and can last up to 24 months [33]. It encompasses structural and biochemical changes in the heart and is generally divided into early inflammatory, intermediate, and late scar maturation phases [32, 34–36]. The early phase lasts up to 72 h post-MI and involves adaptive reactions to maintain stroke volume [32]. The intermediate phase lasts from 72 h to approximately 28 days, during which key processes of dead cardiomyocyte phagocytosis and initial fibrosis occur. This is a critical period for clearing the infarction area of dead cells and initiating repair processes. The significance of this phase for subsequent heart dysfunction should also be emphasized. The late phase extends from one month to up to 24 months [35–37].

Heart muscle deformation occurs due to myocyte stretching, which can cause pathological changes such as myocyte hypertrophy, apoptosis, and changes in the extracellular collagen matrix (ECM) with fibrosis. The late phase begins a month after the ischemic event and includes potentially reversible structural and biochemical changes. Hypertrophy and dilation occur in non-infarct areas as an adaptive response to increased wall stress [34]. This impacts the progress and outcomes of the rehabilitation process. The earlier the rehabilitation process is started, the more effectively the heart muscle will be able to remodel.

### Heart rate in rest and maximal effort

The level of training is evidenced, among other things, by a reduction in resting heart rate (HR rest). In the studied group of patients, no significant changes in this parameter were observed after 5 weeks of comprehensive cardiac rehabilitation. The lack of change in this parameter may result from the short observation period after MI (admission to rehabilitation averaging 17 days from MI) and the short intensive cardiac rehabilitation cycle (5 weeks) [38, 39]. Other authors confirm that the rehabilitation process led to a significant decrease in HR rest, however, after a longer 12-week (3 exercise training per week) rehabilitation process, compared to our study. Patients in the Elshazy et al. 2018 study were admitted to rehabilitation a month after MI [40]. The lack of HR rest reduction in a short time after MI, compared to the results of other authors where a decrease in HR rest is observed over a longer time after MI, may result from physiological changes in the heart remodeling process after myocardial infarction. In our study, the overlapping physiological processes of heart muscle repair after MI with rehabilitation effects do not result in changes visible in the reduction of resting heart rate within such a short time (average 17 days from MI to admission for 5 weeks of intensive rehabilitation). It would be reasonable to examine the same group at a later time.

The lack of change in resting heart rate (HR rest) and one-minute heart rate recovery (1-minute HRR) observed in our study, while initially appearing as neutral findings, may actually have positive clinical implications in the early post-MI rehabilitation context. Stability in these parameters during the critical cardiac remodeling period (mean 17 days post-MI) can be interpreted as a favorable prognostic sign indicating appropriate exercise loading and absence of heart failure symptoms. While long-term cardiac rehabilitation typically leads to decreased resting heart rate after 6–9 months of systematic training, the stability of HR rest and HRR in our short-term, early intervention suggests that the cardiovascular system is adapting appropriately to the rehabilitation program without signs of overload. This interpretation aligns with the significant improvements observed in other exercise capacity parameters (MET, 6MWT, HR max), indicating enhanced functional capacity without disrupting the delicate balance of autonomic regulation during this critical recovery phase [32].

The consequence of improved exercise tolerance after completing the cardiac rehabilitation program was a significant increase in maximal heart rate (HR max) in the studied group. In our previously conducted studies, we evaluated patients after 3 (mean 74 days) and 6 (mean 167 days) months after MI, admitted to a 5-week rehabilitation cycle conducted in the same manner as in this study, where HR max significantly increased. Patients were admitted to cardiac rehabilitation later after MI than in the current study [38]. In contrast to our results, some authors did not observe a significant increase in HR max after the rehabilitation period [40]. The increase in HR max is most likely due to the heart performing more work by overcoming greater load during the final exercise test, as illustrated by the results obtained in a higher MET.

### Heart rate recovery

It has been shown that impaired heart rate recovery (to resting values) is associated with increased risk of cardiovascular and all-cause mortality. A decrease of 15 to 20 beats per minute in the first minute of recovery is typical for healthy individuals [42].

In our study, after a 5-week intensive (5 training sessions/week) cardiac rehabilitation program, we did not observe statistically significant changes in the 1 min HRR parameter. However, another cohort study conducted on 285 individuals who completed 5 to 24 training sessions over 8 weeks of cardiac rehabilitation showed that all patients demonstrated an increase in heart rate recovery, regardless of the number of completed sessions, with a significant correlation between heart rate recovery increase and the number of completed sessions. The authors in this study did not specify the time elapsed between MI and admission to cardiac rehabilitation [41]. Other authors show that heart rate recovery significantly improved in 6-week and 12-week programs compared to baseline values (*p* < 0.001). However, when comparing both programs at the end, heart rate recovery was slightly faster in the 6-week program (26.5 ± 6.78 compared to 23.17 ± 6.12 beats per minute), although it did not reach statistical significance (*p* = 0.051). In this study, patients were admitted to the rehabilitation process 6 months after MI [14].

### Changes in BP

According to scientific research, systolic blood pressure (SBP) during peak exercise may decrease after cardiac rehabilitation (CR) in patients after myocardial infarction (MI) [38, 40, 43, 44]. It should be noted that in studies where a decrease in peak SBP was observed, observations were made at a longer time from MI occurrence than in our study. It should be emphasized that the reduction in peak diastolic blood pressure observed in our study, although statistically significant (*p* = 0.04), represents a relatively small absolute change of 1.47 mmHg (from 80.03 ± 7.32 to 78.56 ± 7.44 mmHg). While this result suggests some hemodynamic adaptation during exercise, its clinical significance should be interpreted cautiously. The modest nature of this change may be related to several factors, including the short duration of the rehabilitation program (5 weeks), the early post-infarction stage (mean 17 days), when other intensive adaptive processes associated with cardiac remodeling are occurring in the body, and the complex mechanisms of blood pressure regulation. In contrast to the relatively minor changes in blood pressure parameters, more significant improvements were observed in other exercise capacity indicators, such as MET, 6MWT distance, and reduction in subjective perception of fatigue on the Borg scale, suggesting that improved exercise tolerance may precede more pronounced hemodynamic changes. Assessment of the long-term impact of early cardiac rehabilitation on blood pressure parameters requires further research with a longer observation period.

### Changes in exercise parameters

During physical exercises in the cardiac rehabilitation program, adaptive processes are activated, especially when exercises are systematically repeated while simultaneously increasing their intensity and volume. A key factor triggering this mechanism is properly applied exercise loads, which condition the improvement of the body’s fitness. In response to increased aerobic activity, heart stroke volume increases and skeletal muscle capillary density improves [45–47].

In this study, already after 5 weeks of completing the cardiac rehabilitation program, which is approximately 7.5 weeks from the implemented clinical treatment of patients after MI, an increase in energy expenditure value expressed in MET was observed compared to parameters before the comprehensive cardiac rehabilitation process. This is the time of the most intensive heart muscle remodeling after an acute coronary event [32–34]. The beneficial effect of comprehensive cardiac rehabilitation on improving MET is also confirmed by studies of other authors [38–40, 54]. The observed MET improvement of 2.22 units (from 6.28 ± 1.98 to 8.50 ± 2.64) significantly exceeds established thresholds for clinically meaningful functional gain. Evidence indicates a 19% lower risk of cardiovascular death per 1-MET improvement in patients with cardiovascular disease [48]. Our observed improvement of 2.22 METs therefore translates to potentially substantial prognostic benefits, particularly as participants progressed from a level of 6.28 METs to 8.50 METs, which may represent a transition from higher to lower risk categories for future cardiovascular events.

Our research results show that the resting index based on the double product reserve (DPr) increased after the 5-week comprehensive cardiac rehabilitation process in the entire studied group. This is the only parameter indicating greater cardiovascular system work (HR max x SBP peak) to overcome the treadmill load resulting from the higher energy expenditure expressed in MET in our research group. It should therefore be noted that the MET load parameter increased significantly, so the cardiovascular system had to overcome a greater load, and consequently, the DPr parameter increased, and the 5-week training duration might still be insufficient to achieve full cardiovascular system adaptation to the applied physical exertion [38, 39]. If the time of comprehensive cardiac rehabilitation were extended to a minimum of 3 months, it could influence not only the increase in the MET parameter but also simultaneously reduce DPr. Our early initiation of rehabilitation (on average 17 days after MI) falls during the period of most intensive cardiac remodeling following an acute coronary event [32–34], which may beneficially affect this process. It has been demonstrated that early rehabilitation interventions can directly influence remodeling processes by modifying the expression of matrix metalloproteinases and limiting unfavorable left ventricular dilation. Furthermore, our results show that after just 5 weeks of intensive rehabilitation, changes in other analyzed parameters (HR max, 6MWT, and Borg scale score) indicate significant improvement in physical fitness [49, 50].

### Changes in 6MWT

In this study, after completing a 5-week comprehensive cardiac rehabilitation program, a significant increase in 6MWT distance of approximately 52 m was observed. Studies by other authors also showed a significant increase in 6MWT distance [54], by 20% (85 m) [51], or 29.8% [52] in patients undergoing a 12-week cardiac rehabilitation program. A meta-analysis [53] demonstrated that the 6MWT distance improved by an estimated mean of 60.43 m after cardiac rehabilitation.

### Changes in Borg scale

In our study, we observed a statistically significant reduction in Borg scale values from 14.06 (± 1.90) to 13.05 (± 0.92) (*p* = 0.0001) after a 5-week comprehensive cardiac rehabilitation program. This change indicates an improvement in patients’ subjective perception of fatigue during similar or greater physical exertion, which may demonstrate the effectiveness of the applied rehabilitation protocol. Our results are consistent with observations by Elshazly et al. (2018) [40].

It is worth emphasizing that the latest research by Nichols et al. (2020) points to a complex relationship between improvement in the Borg scale and actual physiological changes. The authors note that subjective improvement in perceived exertion can occur even with moderate changes in aerobic parameters, suggesting multi-factorial mechanisms of exercise adaptation. In the context of our study, this confirms the value of even short-term rehabilitation programs that can significantly improve exercise tolerance at the patient’s perception level [55].

The current expert position of the European Society of Cardiology, presented by Zaree et al. (2023), emphasizes the importance of subjective exercise assessment parameters, including the Borg scale, as significant indicators of cardiac rehabilitation effectiveness. The authors suggest that reducing Borg scale values at the same or greater load can be one of the key intervention effectiveness indicators, particularly in the context of improving quality of life and returning to daily activities [56].

The latest guidelines on cardiac rehabilitation [57] indicate the crucial role of the Borg scale in personalizing training protocols. The authors emphasize that monitoring changes in exercise perception allows for precise adjustment of training intensity to the patient’s individual capabilities. The reduction in Borg scale values observed in our study may therefore indicate not only improved fitness but also effective individualization of the rehabilitation program.

### Strengths and Limitations of the Article

As a strong point of the article, it is important to emphasize the observation of patients in a short period after MI (average 17 days) until admission to the intensive cardiac rehabilitation process (5 weeks, 5 training sessions/week) and observation of improvement in patients’ physical fitness parameters (HR max, DBP peak, MET, 6MWT), which provides a significant contribution to understanding the effectiveness of rehabilitation started quickly after a coronary event. The study provides evidence supporting the effectiveness of rehabilitation programs implemented in outpatient settings, which is important for healthcare organization and cost optimization. It would be reasonable to examine the same group over a longer observation period, which would show whether beneficial changes also occur in the other studied parameters. The absence of a control group is a clear limitation of this study, which restricts the ability to draw causal inferences. We attempted to mitigate potential confounding factors through careful participant selection and statistical adjustments, acknowledge that the lack of sex-stratified analysis represents an additional limitation, particularly given the male predominance (70.7%) in our study population. Sex differences in cardiac rehabilitation response are clinically relevant and should be addressed in future research through adequate representation of both sexes and planned sex-stratified analyses. However, residual bias cannot be ruled out.

## Conclusions

(1) Early cardiac rehabilitation, implemented on average 17 days after myocardial infarction, leads to significant improvement in physical exercise tolerance after just 5 weeks of an intensive training program, confirmed by significant changes in HR max, DBP peak, MET, and 6MWT parameters.
(2) An intensive cardiac rehabilitation program implemented during the most intensive heart remodeling period may favorably influence this process, suggesting the importance of early rehabilitation intervention in preventing adverse remodeling.
(3) A comprehensive cardiac rehabilitation program, including endurance training, resistance training, and elements of education and psychological support, conducted under the supervision of a physiotherapist, ensures significant improvement in physical fitness for individuals after myocardial infarction.
(4) The study results confirm the value of implementing intensive, 5-week cardiac rehabilitation programs in clinical practice as an effective method of improving exercise tolerance in patients after myocardial infarction, even with a relatively short intervention duration.
(5) The obtained results suggest that even shorter but intensive rehabilitation programs can be an effective alternative to longer programs, particularly in the context of limited healthcare system resources.
(6) The results support implementing early, intensive 5-week cardiac rehabilitation programs as effective alternatives to traditional longer programs, particularly in resource-limited systems, and prioritizing rapid referrals and immediate access to rehabilitation services.

## Supplementary Information

Supplementary Material 1

## Data Availability

Data is provided within the supplementary information files.

## Abbreviations

6MWT: 6-minute walk test
BMI: Body mass index
BP: Blood pressure
CR: Cardiac rehabilitation
CSC-Infarct: Coordinated Specialist Care Program - Infarct
DBP: Diastolic blood pressure
DM: Diabetes mellitus
DPr: Double product reserve
ECG: Electrocardiogram
EF: Ejection fraction
ESC: European Society of Cardiology
FITT: Frequency, intensity, time-duration, type of exercise
HR: Heart rate
HRR: Heart rate recovery
LVEF: Left ventricular ejection fraction
MET: Metabolic equivalent of task
NT: Hypertension
SBP: Systolic blood pressure
WHR: Waist-hip ratio
